# The outbreak, response, and reflections on the chikungunya fever epidemic in Foshan City, China

**DOI:** 10.1186/s41256-025-00458-2

**Published:** 2025-11-10

**Authors:** Nuosu Nama, Yujie Ma, Junwen Zhou, Zhuoya Zhao, Yongyue Wei, Yuantao Hao

**Affiliations:** 1 Department of Epidemiology and Biostatistics, School of Public Health, Peking University, Beijing, China; 2 Center for Public Health and Epidemic Preparedness & Response, Peking University, Beijing, China; 3 Key Laboratory of Epidemiology of Major Diseases (Peking University), Ministry of Education, Beijing, China

**Keywords:** Chikungunya, Foshan outbreak, Vector-borne disease surveillance, Emergency response, Early warning system

## Abstract

Driven by climate change, viral evolution, intensified human mobility, and increasing population susceptibility, mosquito-borne diseases are extending their geographic range and public health impact worldwide. The 2025 chikungunya outbreak in Foshan, Guangdong Province, provided a critical real-world assessment of the province’s local surveillance and emergency response system. This commentary aims to elucidate the effectiveness, strengths, and limitations of Foshan’s outbreak response, and to identify lessons for strengthening early warning and vector-borne disease preparedness. Containment was achieved within three weeks through coordinated multi-sectoral emergency activation, rapid case detection, and precision vector control. However, the response also exposed systemic vulnerabilities, including delayed activation of warning mechanisms, limitations in entomological surveillance, and deficiencies in multi-source data integration. Addressing these gaps will require establishing a multi-source, data-driven early warning system; institutionalizing standardized cross-border surveillance and response protocols; expanding genomic and epidemiological research capacity; and strengthening research capacities and workforce resources from a global perspective to enhance global preparedness against emerging mosquito-borne diseases.

## Introduction

Chikungunya virus (CHIKV), an arthropod-borne alphavirus transmitted primarily by *Aedes aegypti* and *Aedes albopictus*, was first identified in Tanzania in 1952 [1]. It has since been endemic in many tropical and subtropical regions. By the end of 2024, over 110 countries had reported locally acquired or imported cases; in the first seven months of 2025, more than 240,000 cases and 90 deaths were reported globally [2].

**Fig. 1:**
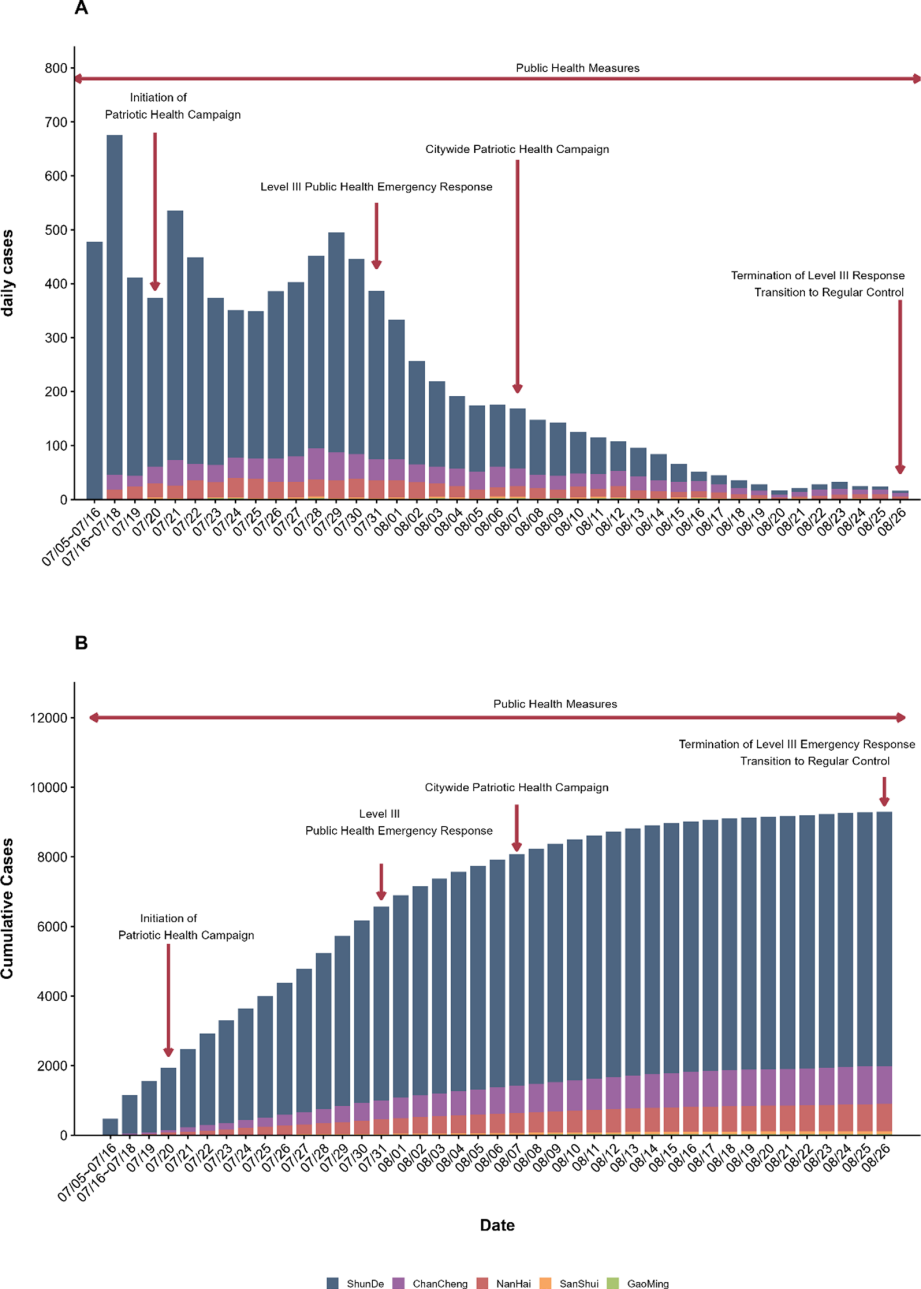
Distribution of cases during the CHIKV outbreak in Foshan, July 5 to August 26. *Note*: Cases represent laboratory-confirmed cases reported by Foshan municipal and district health authorities; under-detection and underreporting may exist

On July 8, 2025, routine public health surveillance in Shunde District, Foshan City, Guangdong Province, identified an imported case of chikungunya fever (CHIKF). By July 15, a cumulative total of 478 laboratory-confirmed cases had been reported. The outbreak subsequently extended from Foshan to parts of the Pearl River Delta and other areas of Guangdong Province. Based on official reports released by the municipal authorities of Foshan and the districts of Shunde, Chancheng, Gaoming, Nanhai, and Sanshui, the epidemic peaked on July 21, when 536 new cases were reported in Foshan in a single day, and the daily incidence continuously declined after July 29 (Fig. 1). By August 8, over 90% of patients had recovered, with no severe cases or fatalities, and without significant public panic or misinformation observed. The majority of cases occurred in Shunde District, with limited secondary clusters in Chancheng and Nanhai District.

In response to the outbreak, local municipal authorities activated the emergency response framework and established an intersectoral epidemic prevention and control command structure. This mechanism coordinated the efforts of municipal health authorities, the Center for Disease Control and Prevention (CDC), urban management, and education departments, facilitating joint prevention and control measures. The Patriotic Health Campaign, a long-standing national initiative promoting environmental sanitation and vector control, was mobilized for large-scale community engagement. A Level III public health emergency response was launched on July 29, followed by a one-week citywide “Adult Mosquito Eradication Special Action” starting July 31. Updated technical guidelines: the *Diagnosis and Treatment Protocol for Chikungunya Fever* (2025 Edition) and the *Technical guidelines for the prevention and control of Chikungunya Fever (2025 Edition)*, were issued to standardize case management, improve coordination between clinical and public health sectors, and guide community-level interventions.

## Advantages and shortcomings in the response

Over the past decade, Guangdong Province has developed an integrated framework for controlling infectious diseases that combines pathogen-specific diagnostics, syndromic and vector surveillance, early warning systems, and rapid response mechanisms. The 2025 Foshan outbreak served as a real-world stress test of this system, which successfully achieved containment within approximately three weeks.

The response demonstrated several strengths, many of which stemmed from institutional and technological advances accumulated during the COVID-19 pandemic. At the government level, the rapid coordination framework developed during COVID-19 was quickly adapted to chikungunya control, with clear reporting lines and defined responsibilities across agencies. Primary healthcare facilities adhered to the “early reporting, isolation, and management” principle embedded in China’s national infectious disease response framework, ensuring timely case detection and containment. Remarkably, a sentinel primary care physician identified a cluster of similar symptoms and promptly reported it, shortening the interval from the first suspected case to CDC awareness from 30 days during the 2010 Dongguan outbreak to just 11 days in 2025—a substantial improvement.

In addition, technological innovations enhanced operational efficiency. The electronic sentinel platform, integrating automated mosquito traps, detection sensors, and machine-learning algorithms, provided weekly mosquito density updates, enabling precise targeting of interventions. Unmanned aerial vehicle (UAV) spraying and sterile insect technique (SIT) releases were deployed in line with WHO-recommended integrated vector management approaches. The rapid expansion of testing capacity reduced diagnostic delays, while spatiotemporal tracing facilitated the timely disruption of transmission chains. Public communication, such as daily epidemiological updates and mosquito forecasts and risk maps published across multiple platforms, improved public engagement, personal protective awareness, and behaviors among residents.

More importantly, community-level interventions played a critical role in epidemic control. Through the Patriotic Health Campaign, large-scale household and neighborhood-level initiatives, such as eliminating stagnant water and managing stored water, effectively reduced vector breeding sites. These interventions not only lowered mosquito density but also strengthened community-level disease awareness and self-protection behaviors. Notably, these strategies were not unique to Foshan. During the 2010 chikungunya fever outbreak in Dongguan, when medical resources and technologies were far less developed, community mobilization through the Patriotic Health Campaign was equally central to containment. This illustrates that broad-based community engagement represents a universally applicable and sustainable strategy across different settings. Evidence from outside China supports this conclusion: a mixed-methods study in Curaçao, Latin America, found that community involvement, by raising awareness and reducing mosquito breeding sites, was highly effective in mitigating mosquito-borne disease transmission [3].

However, the outbreak highlighted important areas for improvement, especially in the mosquito-borne disease prevention and control system. First, response timeliness. While the interval from the first suspected case to CDC awareness was reduced drastically compared with Dongguan in 2010 (from 30 to 11 days), there was still a seven-day lag between CDC awareness (July 8) and issuance of an official risk warning (July 15). This delay suggests a disconnect between early warning signals and emergency activation, warranting closer analysis of decision thresholds, risk communication protocols, and interagency information flow. Second, surveillance limitations. Entomological surveillance still relied heavily on the Breteau Index, which requires manual larval surveys, limits temporal resolution, and does not integrate climatic variables (e.g., temperature, humidity) that influence vector proliferation [4]. Third, cross-border intelligence gaps. Effective monitoring of diseases such as chikungunya requires integrating human population data, vector surveillance, climatic factors, and epidemiological information from neighboring countries. China lacks of an established reporting and feedback mechanism for arbovirus activity in neighboring endemic regions, particularly in Southeast Asia, limiting early detection of importation risk [5].

Overall, while Guangdong’s response exhibited strong adaptive capacity and technological integration, addressing the surveillance–response gap, modernizing entomological monitoring, and strengthening international multi-source data sharing and monitoring will be essential to achieving sustained control of emerging arboviruses under increasingly favorable ecological and climatic conditions. These challenges are not unique to chikungunya but represent systemic vulnerabilities that could similarly affect dengue, Zika, and other *Aedes*-borne diseases.

## Lessons learned and policy recommendations

Despite successful containment, the Foshan outbreak demonstrates that post-epidemic vigilance remains critical, particularly in the context of imported-case risk and *Aedes*-driven seasonal resurgence, and the potential for geographic spread to surrounding municipalities. The observed case distribution pattern suggests an outward diffusion tendency within the province, highlighting the need for heightened prevention and control in adjacent Class I risk regions and neighboring provinces with high ecological suitability for *Aedes* proliferation. The WHO July 22 global alert [6], coupled with the forecast of continued high mosquito activity in Guangdong, further underscores the importance of post-epidemic vigilance, continued surveillance, and sustained community participation in vector control to prevent secondary transmission or reintroduction events.

In the short term, several actionable recommendations emerge. First, case detection and activation mechanisms should be strengthened by refining thresholds, tightening reporting timelines, and ensuring that local CDC alerts promptly trigger risk communication and early interventions. Emergency response capacity must be reinforced through clear protocols and regular readiness verification [7]. A unified, tiered national response guideline—detailing activation thresholds, reporting timeframes, inter-agency command hierarchies, and logistics pipelines down to township and community levels—should be codified and operationalized. Second, the existing sentinel surveillance network should be optimized by extending its coverage to peri-urban and semi-rural high-risk areas, with deployment of automated oviposition traps and integration of rapid viral testing of both mosquito pools and human samples to shorten the detection-to-response interval. Third, community-based interventions remain the cornerstone of epidemic control. The Patriotic Health Campaign should be institutionalized as a routine, pre-seasonal activity, with consistent messaging and mobilization of residents for source reduction. Annual multi-agency tabletop exercises and simulation drills could also help stress-test outbreak response protocols, ensuring that rapid response teams remain trained, resourced, and ready for deployment at the first sign of transmission, especially for arboviruses with seasonal potential such as dengue, chikungunya, Zika.

Beyond these immediate priorities, several strategic and long-term objectives should be pursued to strengthen systemic resilience. A top priority is to develop a multi-source, data-driven early warning system for vector-borne diseases [8], fully incorporating the *One Health* framework and operationalizing the WHO’s *Global Vector Control Response 2017–2030*. Such a platform would integrate global arboviral epidemiology data, cross-border mobility analytics, high-resolution meteorological and climate forecasts, entomological indices, and localized sero-immunity profiling to generate a comprehensive risk picture. Coupled with AI-assisted analytics, this system could support near real-time outbreak prediction, scenario modeling, and evidence-based decision-making, allowing public health authorities to intervene earlier and more precisely.

Another priority is establishing an operational cross-border intelligence network, particularly with Southeast Asia, where the risk of vector-borne disease importation is high. Strengthening international and cross-border collaboration is essential, not only to harmonize case definitions, diagnostic platforms, control standards, and vector surveillance protocols, but also to establish a joint early warning and feedback mechanism modeled on existing influenza and poliomyelitis surveillance networks. Building on this, regional capacity-building initiatives could focus on establishing centers of excellence for vector-borne disease research, standardized training for clinicians and entomologists, and joint international exercises to strengthen preparedness. Real-time data-sharing platforms and international exchange programs would further promote globally coordinated response capabilities.

In the longer term, sustained investment in research and innovation will be critical [7, 9]. Priorities include accelerated phase II/III trials of candidate vaccines, real-world effectiveness evaluations, and development of targeted immunization strategies for high-incidence districts. Establishing a national genomic surveillance database for vector-borne viruses would enable real-time monitoring of viral evolution, phylogeographic spread, and resistance-linked genomic markers [10]. Such a database would directly inform vaccine strain selection, vector control strategies, and risk communication. Therapeutic research should focus on antiviral treatments, refining symptomatic care protocols, and managing post-viral sequelae such as chronic arthralgia. Finally, technological innovation in vector control should advance toward intelligence and precision by expanding electronic sentinel networks, deploying AI-assisted smart traps, leveraging Wolbachia-based biocontrol, scaling UAV-based adulticide dispersal, and implementing sterile insect technique (SIT) programs to improve efficiency and reduce labor dependence.

Together, these short-term actions and long-term strategies provide a comprehensive framework to enhance early detection, accelerate response, and build durable resilience against arboviral threats.

## Conclusion

Driven by the growing threat of mosquito-borne disease due to climate change, viral evolution, and increasing population susceptibility, Foshan’s experience with the 2025 chikungunya outbreak provides a valuable case for evaluating outbreak preparedness and response.

The outbreak demonstrates that rapid containment is achievable when surveillance, research, community engagement, and governance operate as an integrated and adaptive system. However, the episode also reveals structural vulnerabilities, particularly in early warning and response linkages, dynamic entomological risk assessment, and cross-border intelligence exchange, which could hinder timely containment of future arboviral threats. Moving forward, institutionalizing a multi-source rapid-response early warning system, strengthening inter-jurisdictional and international coordination, embedding community engagement into formal prevention frameworks, and sustaining investment in technological and human capital will be essential. By aligning these strategies with global health frameworks and maintaining operational readiness during inter-epidemic periods, Guangdong, and China more broadly, can not only reduce domestic chikungunya risk but also contribute substantively to global preparedness against the escalating threat of emerging *Aedes*-borne diseases.

## Data Availability

All data and sources referenced in this commentary are publicly available from published literature, official reports, and government or WHO sources.

## Abbreviations

CHIKV: Chikungunya virus
CHIKF: Chikungunya fever
WHO: World Health Organization
SIT: Sterile insect technique
CDC: Center for disease control and prevention
COVID-19: Coronavirus disease 2019
UAV: Unmanned aerial vehicle
AI: Artificial intelligence
